# Physics constrained graph neural network for real time prediction of intracranial aneurysm hemodynamics

**DOI:** 10.1038/s41746-026-02404-z

**Published:** 2026-02-06

**Authors:** Vincent Lannelongue, Paul Garnier, Pablo Jeken-Rico, Aurèle Goetz, Philippe Meliga, Yves Chau, Elie Hachem

**Affiliations:** 1 Mines Paris - PSL, University Centre for Material Forming (CEMEF) CNRS, Sophia Antipolis Cedex, France; 2 Interventional Neuroradiology Department, Nice University Hospital, Nice, France

**Keywords:** Biomedical engineering, Fluid dynamics, Computational models, Machine learning, Predictive medicine

## Abstract

Intracranial aneurysms (IAs) are life-threatening vascular conditions requiring accurate risk assessment to guide treatment. Hemodynamic biomarkers such as wall shear stress and oscillatory shear index are promising predictors of rupture risk but remain underused clinically due to the high computational cost of traditional CFD methods. We propose a physics-constrained graph neural network (GNN) framework trained on high-fidelity CFD data to predict full 3D, time-resolved hemodynamic fields throughout the cardiac cycle. Our model incorporates enhanced node features and physics-based constraints to capture complex spatio-temporal flow behavior in near real time. It generalizes to varying inflow conditions and unseen patient-specific geometries with no fine-tuning. Additionally, we release a benchmark dataset of 105 patient-derived aneurysm geometries with CFD fields to support the machine learning (ML) community. This is the first GNN model applied to transient 3D aneurysmal flow prediction, paving the way for rapid, AI-driven hemodynamic analysis toward risk stratification and treatment planning.

## Introduction

Intracranial aneurysms (IAs) are pathological dilations of cerebral arteries, most frequently found in the Circle of Willis, that affect approximately 2–3% of the population. These aneurysms pose a significant clinical challenge due to their potential to rupture, resulting in subarachnoid hemorrhage, a condition with a case fatality rate over 30%^1,2^. Current clinical decisions regarding aneurysm management rely heavily on static, macroscopic metrics such as size, shape, and location, along with patient-specific factors like age and hypertension history^3^. However, these metrics have limited reliability in predicting rupture risk, often leading to inconclusive or contradictory assessments^4^.

Recent advances in computational methods and imaging technologies have enabled the modeling of aneurysmal blood flow, highlighting hemodynamic biomarkers such as wall shear stress (WSS), oscillatory shear index (OSI), and intra-aneurysmal pressure gradients as key determinants of rupture risk^4^. Computational fluid dynamics (CFD) has been instrumental in providing high-fidelity, patient-specific hemodynamic simulations^5,6^. Despite its utility, the prohibitive computational cost, time-intensive setup, and specialized expertise required for CFD limit its integration into clinical workflows, where timely decision-making is critical.

In parallel, artificial intelligence (AI) and machine learning (ML) methods have demonstrated transformative potential across a range of neurovascular interventions, as evidenced by their application in device tracking during endovascular procedures. For instance, real-time AI-assisted platforms like Neuro-Vascular Assist (iMed technologies, Tokyo, Japan) have improved precision and reduced cognitive load during interventions such as aneurysm coiling and liquid embolization^7,8^, demonstrating the clinical feasibility of AI-driven decision support. Inspired by these successes, researchers have begun exploring ML-based surrogates for CFD, aiming to achieve accurate, near-instantaneous predictions of hemodynamics^9^. Traditional ML approaches have largely relied on convolutional neural networks (CNNs) due to their effectiveness in image-based tasks. While CNNs have shown promise in predicting certain hemodynamic parameters from 2D or simplified 3D data^10–12^, their reliance on structured grids makes them ill-suited for the complex, unstructured nature of aneurysm geometries. This limitation is particularly patent when attempting to model the intricate, three-dimensional flow patterns critical to understanding aneurysm pathophysiology.

Graph neural networks (GNNs) provide a compelling alternative by leveraging the graph-like structure of CFD domains, enabling the direct processing of irregular, non-Euclidean data. Going beyond point cloud-based methods like PointNet and PointNet++^13,14^, which have been tested on simplified geometries and stationary hemodynamics predictions^15,16^, GNNs rely on message passing mechanisms to iteratively update unstructured graphs and efficiently learn local patterns.

Recent studies have demonstrated the efficiency of GNNs in predicting 2D time-dependent flows, steady-state 3D flows, and external flows around simple geometries, particularly in physics-driven tasks involving partial differential equations and numerical simulation^17–22^. To the best of our knowledge, while GNNs have been used in biomedical blood flow and hemodynamic studies^23–28^, their application to time-dependent 3D blood flow predictions with intricate vortex and swirling patterns within IAs remains an unexplored frontier.

This work introduces a novel framework that integrates CFD-trained, physics-constrained graph neural networks (GNNs) to predict full 3D, time-resolved hemodynamic fields for IAs throughout the cardiac cycle. To enhance the accuracy of capturing complex spatio-temporal dynamics, the framework incorporates physics-based embedding laws and constraints directly into the learning process. Additionally, it leverages detailed node feature enhancements within the graph representation, enabling precise modeling of intricate hemodynamic variations. Therefore, the proposed learning-prediction framework achieves a balance between computational efficiency and accuracy, addressing the need for high-fidelity simulations while meeting the immediacy required for clinical applications. To further validate the generalizability of our approach, we perform two inference experiments on out-of-distribution cases: new inflow profiles unseen during training, and four fully patient-specific aneurysm geometries. These results highlight the model’s robustness and its potential as a transferable foundation for future clinical extensions. Finally, to support the broader research community, we introduce a comprehensive dataset comprising 105 patient-specific aneurysm geometries coupled with high-resolution CFD simulations, referred to as BenchAnXplore. Unlike existing datasets, which often suffer from limited size or heterogeneity, this dataset enables standardized evaluation of ML models in aneurysm modeling.

This work lays the foundation for future clinical applications with significant potential impact. Indeed, real-time prediction of hemodynamics could support future advances in rupture risk assessment, enable more personalized treatment planning^29^, assist in optimizing interventions such as flow diverters or coiling^30^, and accelerate long-term simulations of IAs healing^31^. Moreover, the introduction of a comprehensive benchmark dataset helps address a major limitation in the field, the scarcity of large-scale, high-quality training data for ML models. By integrating modern ML architectures with domain-specific knowledge, this study contributes a foundational step toward a new generation of AI-assisted neurovascular modeling. It lays the groundwork for future innovations in clinical decision support, with the long-term goal of improving outcomes for patients with IAs.

## Results

### Dataset

**Fig. 1:**
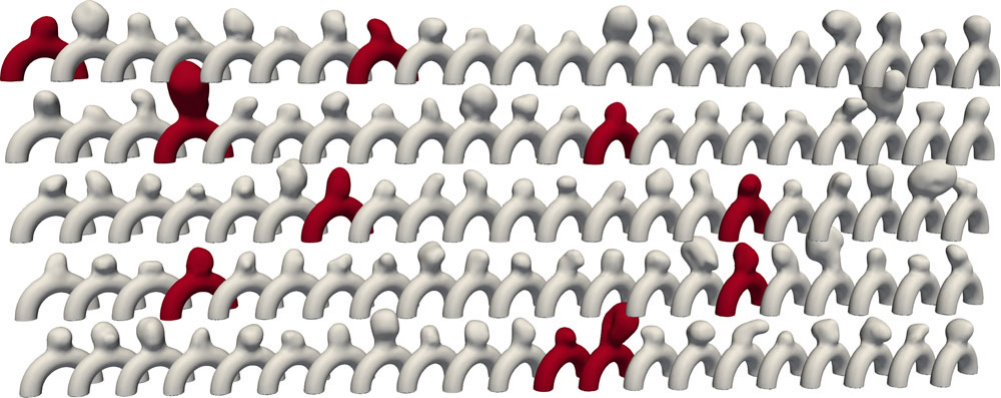
Dataset of 105 semi-idealized patient-specific geometries^33^ with 10 highlighted test cases.

The dataset consists of 105 aneurysm 3D meshes, created by intersecting an ideal half torus with aneurysm bulges extracted from patient cases in the *IntrA* dataset^32^, following Goetz et al. methodology^33^. We refer to them as semi-idealized geometries, as they combine an idealized toroidal parent artery with a patient-specific aneurysm bulge, as illustrated in Fig. 1.

These chosen geometries represent sidewall aneurysms located on the internal carotid artery (ICA), particularly near the ICA siphon. This choice is motivated by its clinical representativeness: the ICA siphon region is a common site for aneurysm formation and is frequently modeled in hemodynamic studies due to its characteristic curvature^34^. Several prior works have highlighted the ICA’s susceptibility to aneurysm development at curved segments due to altered flow patterns and WSS concentrations^35^. Notably, the siphon’s curvature is often approximated as toroidal, making the torus-shaped backbone both anatomically justified and reproducible across computational settings.

The bulges range from 3 to 9 mm in diameter, covering diverse pathological shapes, and the selected vessel diameter in our dataset is 4 mm, which aligns with clinical measurements of the ICA in adult patients typically ranging from 3 to 6 mm^36^.

We generated the **BenchAnXplore** dataset following the meshing, coarsening, and simulation processes further detailed in the Methods section, and obtained 105 aneurysm geometries, with 80 simulated timeframes for each of these cases. This dataset is now made available as a benchmark case for AI IAs applications.

### Training

We trained and compared four models to evaluate the impact of node feature enhancement and physically-informed improvements separately. To ensure a fair comparison, all models were trained using the same configuration. All MLPs (multilayer perceptrons) used for encoders, decoders, and update functions within the GN blocks have two hidden layers of 128 neurons each and use a ReLU activation function. We used 15 rounds of message passing, bringing the total number of parameters to approximately 3 million.

**Fig. 2:**
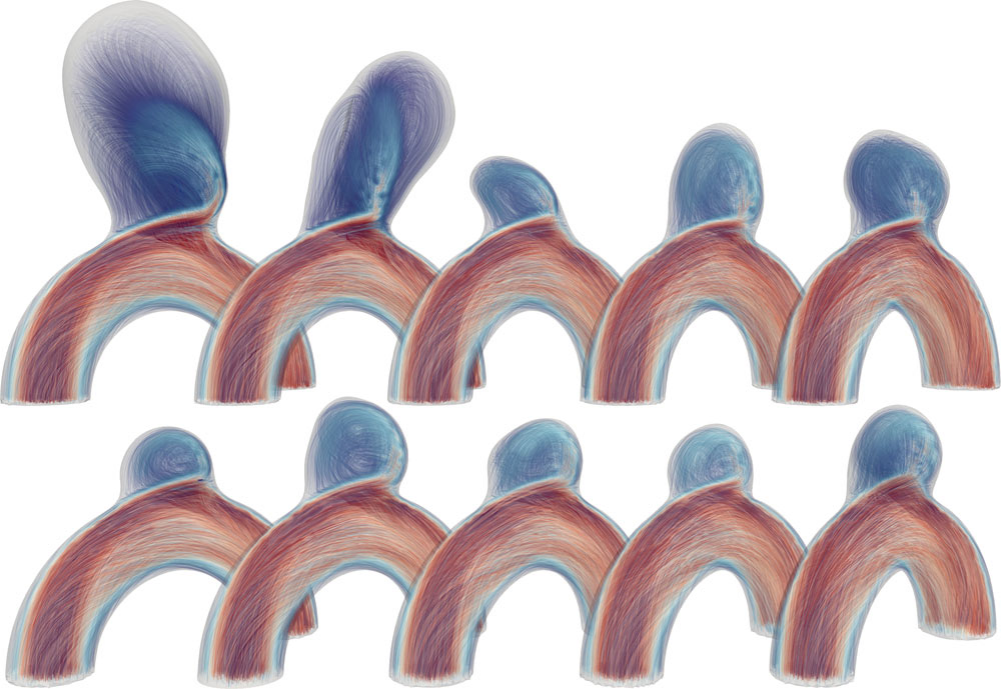
Test set of 10 representative blood flow CFD simulations.

With 105 simulations, each spanning 80 timesteps, we trained on 95 simulations and randomly selected 10 for testing and evaluation (highlighted in red in Fig. 1). This test set gathers a wide variety in shapes and flow characteristics as depicted in Figure 2. Given that our meshes contain in average, 20,000 nodes, this results in over 75 million training tokens. Recall that the number of training tokens is equal to trajectories * timesteps * data points.

We trained for 20 epochs, e.g., 144k training steps, using a fixed learning rate of 10^−4^ for 16 epochs followed by 4 epochs with exponential decay to reach a minimum learning rate of 10^−7^. Training was conducted on L4 NVIDIA GPUs, taking approximately 20 h, with a slight (~5%) increase for the physically informed network due to gradient computations.

#### Boundary conditions

To handle boundary conditions over the domain, we added a node type as an input feature to separate different training behaviors according to the considered nodes. We fixed a null velocity for the wall nodes and did not compute loss nor performed learning onto these nodes. The velocity at the inlet nodes was fixed; we imposed the same pulsatile parabolic inflow profile as in the ground-truth CFD simulations. All other nodes of the fluid domain, outflow included, were used to train the network.

#### Training noise

Our models are trained for 1-step prediction tasks but are intended to be used in a “rollout" scenario, where they iteratively use their own predictions to generate an entire simulation. In such scenarios, the model’s input data can deviate from the training distribution due to errors introduced in previous steps. This deviation amplifies errors over successive iterations, resulting in cumulative inaccuracies throughout the simulation. Following the same strategy as^37^, we introduced noise to all our dynamical variables during training to enhance the robustness of our models.

#### Timestep

To simplify training and eliminate temporal ambiguity, we fixed the training timestep to 0.01 s, which is coarser than the CFD solver’s timestep of 0.001 s. This was done intentionally to reduce sequence length while maintaining temporal coherence. As such, the model is not required to generalize to arbitrary time horizons. While we did not test interpolation across multiple timestep sizes, the current design is adequate for clinical use cases where a consistent frame rate is expected.

### Numerical performance

**Fig. 3:**
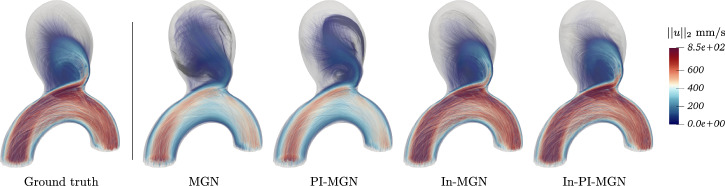
Rollout predictions at time *t*_2_ for all trained models.

We evaluated all four models on the full test set for both next-step and rollout prediction with 1-step and 50-step Root Mean Square Errors (RMSE) as described in the Methods section; mean errors over all test cases are given in Table 1. While all models perform well on next-step prediction, as shown by the 1-step error in Table 1, significant differences emerge in rollout prediction in particular at time *t*_2_ as clearly highlighted in Fig. 3.

**Table 1:** Model configurations and performance

Model	Input features	Loss function	1-RMSE	50-RMSE	50-RMSE SD
MGN	*u*	${{\mathcal{L}}}_{data}$	1.11	50.51	1.43
PI-MGN	*u*	${{\mathcal{L}}}_{PI}$	*1.06*	55.62	1.27
In-MGN	*u*, *du*/*dt*, inflow	${{\mathcal{L}}}_{data}$	1.09	*9.22*	1.47
In-PI-MGN	*u*, *du*/*dt*, inflow	${{\mathcal{L}}}_{PI}$	**0.85**	**7.58**	1.02

Errors averaged on the test set over the whole geometry; next step prediction (1-RMSE) and rollout predictions (50-RMSE) errors are given in mm/s. The standard deviation (SD) of the 50-RMSE across the test set cases is also displayed. Best results are displayed in **bold**; second best in *italic.*

Indeed, the In-MGN and In-PI-MGN models produce high-quality rollouts, with the 50-step error for In-PI-MGN being more than six times lower than the MGN baseline. In-MGN also performs well on longer predictions, whereas introducing the physical loss alone does not improve long-term prediction performance. Both MGN and PI-MGN models converge for single-step predictions but struggle with error accumulation in rollout predictions.

**Fig. 4:**
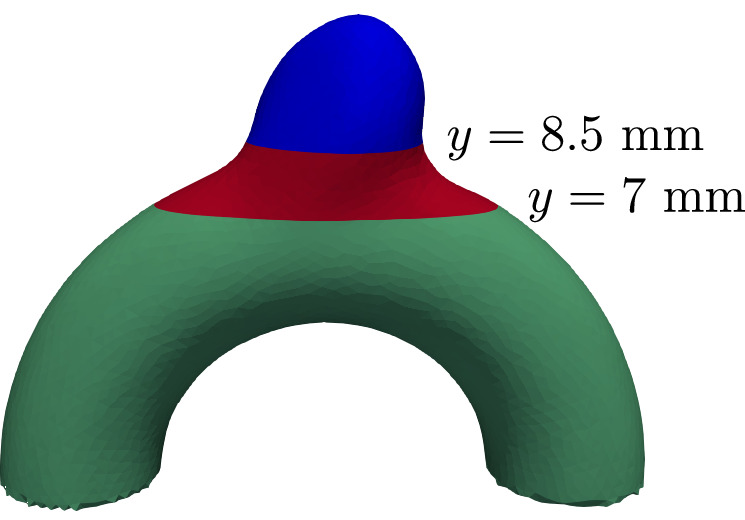
Split of our geometries at planes *y* = 8.5 mm and *y* = 7 mm to evaluate RMSE metrics in three anatomically regions: the aneurysm bulge (blue), the neck (red), and the parent artery (green).

Because all geometries share the same parent artery, averaging the errors over the entire domain may obscure localized variations in model performance. To better assess the predictive accuracy of each model, we computed the 1-step and 50-step RMSEs separately across three anatomically distinct regions: the aneurysm bulge, the neck, and the distal segment of the parent artery (see Fig. 4). The results for all four models are summarized in Table 2, confirming the same overall tendencies observed in the global error metrics.

**Table 2:** 1-RMSE and 50-RMSE in mm/s averaged across the test set on the different regions of the geometries

Model	Bulge 1-RMSE	Neck 1-RMSE	Artery 1-RMSE
MGN	1.22	1.63	0.91
PI-MGN	1.14	1.31	0.92
In-MGN	1.11	1.47	0.83
In-PI-MGN	**0.68**	**1.16**	**0.74**
**Model**	**Bulge 50-RMSE**	**Neck 50-RMSE**	**Artery 50-RMSE**
MGN	38.16	62.25	51.77
PI-MGN	44.88	66.01	57.87
In-MGN	18.28	10.66	**4.62**
In-PI-MGN	**11.87**	**8.38**	4.66

Best results are displayed in **bold**; second best in *italic*.

To assess the individual impact of acceleration and inflow information on model performance, we conducted an ablation study as part of our experiments. The results showed that adding inflow features alone improved both training convergence and long-term rollout predictions, whereas adding acceleration without inflow features did not lead to model convergence. This suggests that local temporal history is insufficient for accurate prediction in the absence of global context. The combination of both inflow and acceleration features yielded the best performance in terms of error metrics and qualitative agreement with CFD fields.

**Fig. 5:**
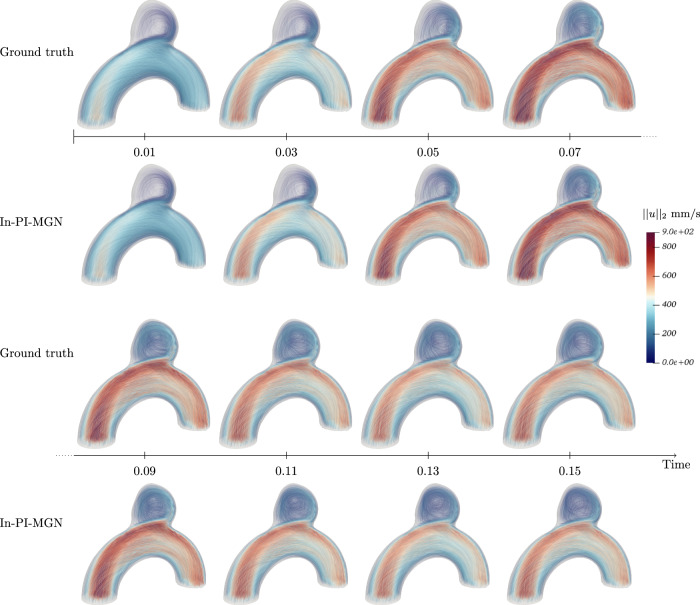
Blood flow during systole for Case B, showing predictions closely matching simulations throughout the acceleration phase.

**Fig. 6:**
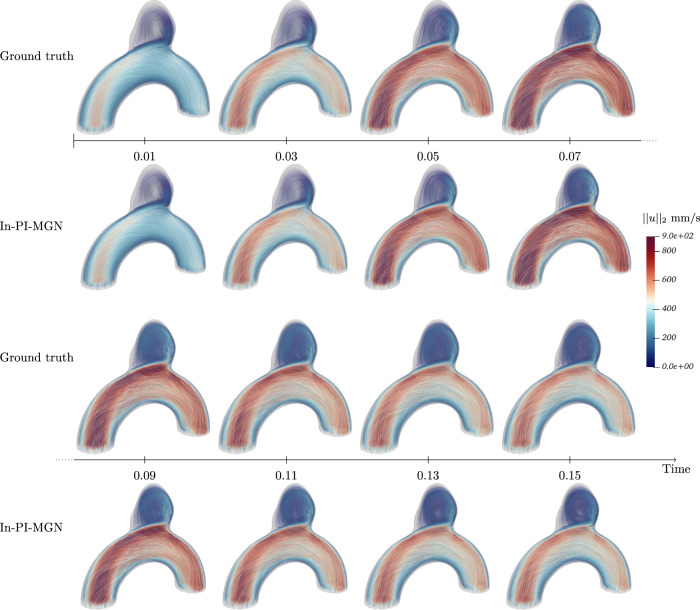
Blood flow during systole for Case C, demonstrating accurate predictions in a larger, more asymmetrical aneurysm bulge.

**Fig. 7:**
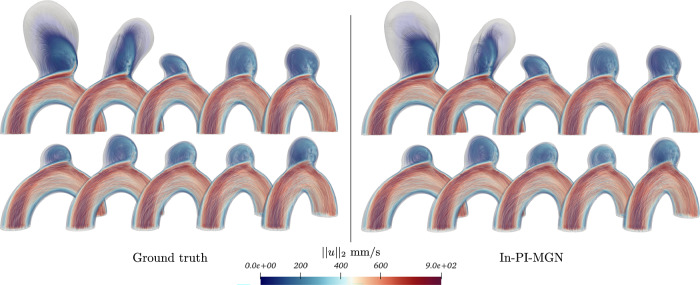
Velocity fields comparison for the whole test set at time *t*_2_.

Figures 5 and 6 show a temporal analysis of the full geometry volume. It clearly demonstrates that GNN predictions are closely aligned with ground truth CFD simulations, even during the systolic phase, where velocity fluctuations are pronounced. Additionally, the model effectively captures the evolution of recirculation within the aneurysm bulge. To assess the model’s performance across a diverse range of flow characteristics and bulge shapes, velocity predictions were generated for the entire test set, as illustrated in Fig. 7.

**Fig. 8:**
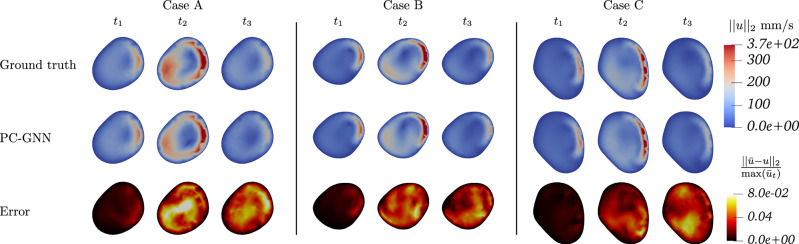
Blood velocity comparison at the aneurysm neck; displayed error is normalized by maximum ground-truth velocity at the given timestep. Maximum error is occurring at high-speed flow regions close to the wall, and remains below 8%.

**Fig. 9:**
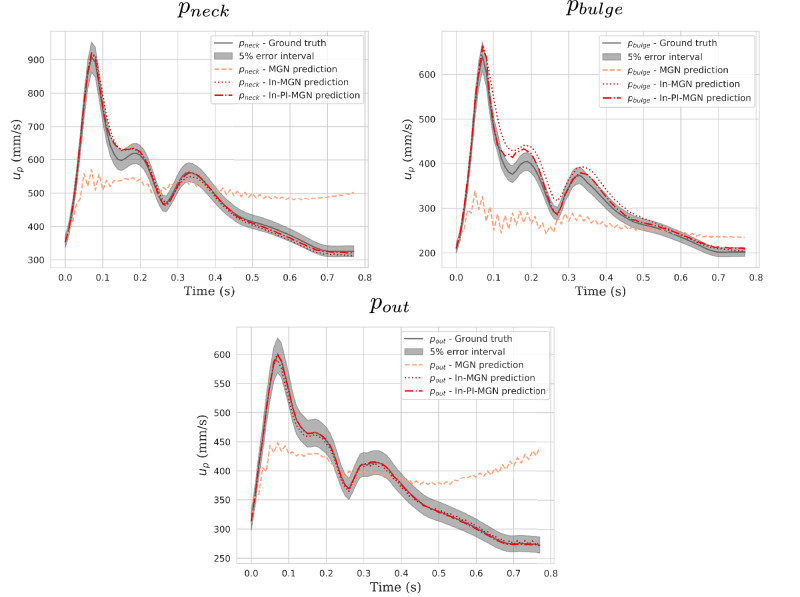
Velocity evolution plotted over time for Case A.

Quantitative comparisons at the aneurysm neck (Fig. 8) demonstrate good agreement in predicted flow dynamics, particularly where high-velocity jets interact with the aneurysm wall. Velocity measurements at evaluation points (Fig. 9) further illustrate model accuracy. While the baseline MGN model deviates significantly from the ground truth after a few timesteps, both inflow-enhanced In-MGN and In-PI-MGN maintain an error margin within 5% for most of the cardiac cycle at *p*_*neck*_ and *p*_*out*_. Predictions at *p*_*wall*_ are less precise for In-MGN and exhibit some delay, whereas In-PI-MGN remains closely aligned with the ground truth, particularly after systole.

**Fig. 10:**
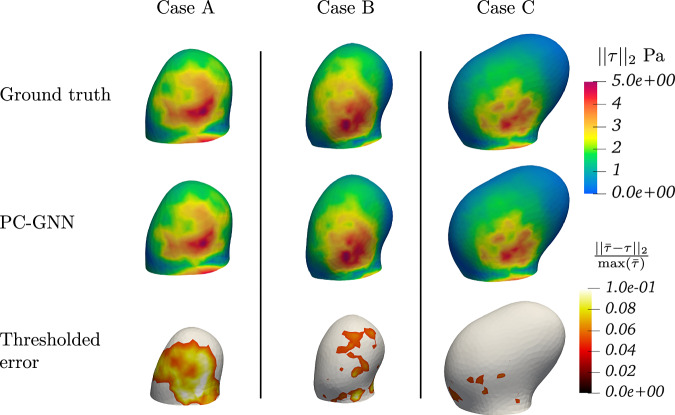
TAWSS magnitude plotted on the aneurysm bulge, and relative error normalized by maximum ground-truth TAWSS. Error is thresholded to mask errors under 5%; maximum local error is at 9%.

**Fig. 11:**
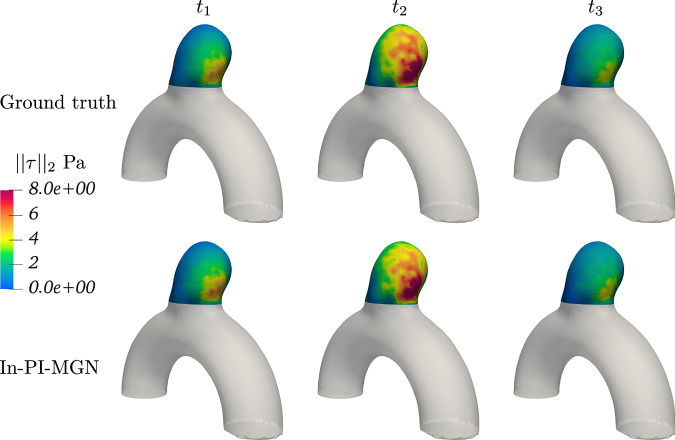
WSS plotted over the aneurysm bulge at different timeframes for Case B.

**Fig. 12:**
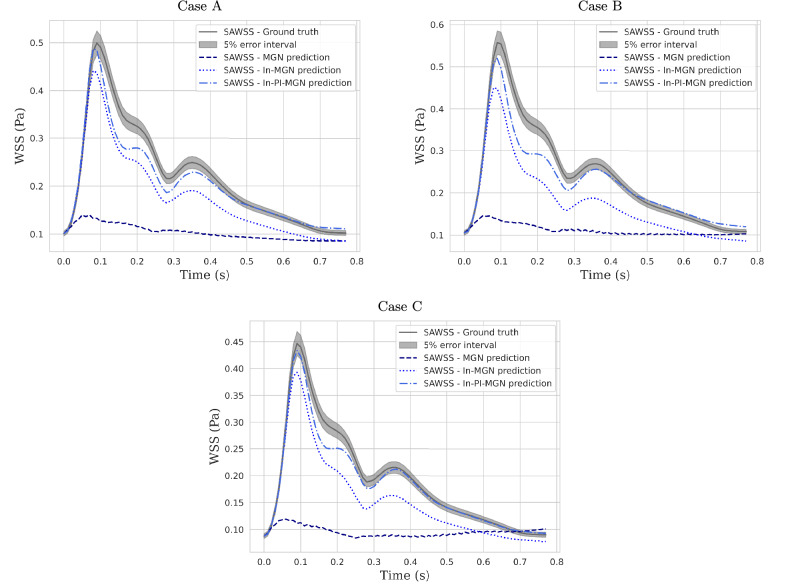
SAWSS computed over the aneurysm bulge for all 3 test cases.

Beyond velocity evaluation, WSS results derived from velocity fields were also analyzed by comparing TAWSS in the aneurysm bulge for the In-PI-MGN model and the ground truth, as shown in Figs. 10 and 11. The prediction error remains within 10% across the entire test set, demonstrating strong agreement between predicted and reference patterns. Finally, instant WSS and space-averaged WSS (SAWSS) were examined in Fig. 12 to track WSS variations throughout the cardiac cycle. The SAWSS curves indicate that while the In-MGN model follows the general trend, the In-PI-MGN model exhibits the closest match to the reference, particularly in capturing peak values and transient dynamics. The WSS plots at our three evaluation timesteps confirm again the accuracy of the model pattern prediction over the cardiac cycle.

**Fig. 13:**
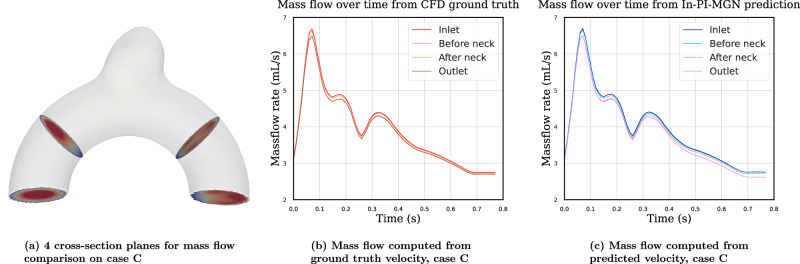
Transient mass flow comparison between CFD ground truth and In-PI-MGN predictions. **a** Case C geometry illustrating the four cross-sectional planes used for mass flow evaluation: inlet, before the neck, after the neck, and outlet. **b** Mass flow rates computed from the CFD ground-truth velocity field at the four cross-sections over one cardiac cycle. **c** Mass flow rates computed from the In-PI-MGN–predicted velocity field at the same cross-sections. Mass flow is reported in mL s⁻¹ as a function of time.

Furthermore, in order to evaluate the ability of the model to conserve mass, we conducted quantitative mass flow analyses at multiple cross-sectional planes along the artery, as displayed in Fig. 13. Specifically, we measured the instantaneous volumetric flow rates at the inlet, the outlet, and at two additional intermediate planes normal to the centerline of the parent artery. These measurements were performed across the full cardiac cycle for both the CFD ground truth and the In-PI-MGN model predictions.

The results are presented in Fig. 13, illustrating the flow rates across the geometry for both the ground truth and the predicted velocity fields. The mean difference between inlet and outlet flow rates for ground truth and prediction are 2.3% and 3.7%, respectively, with maximums across the cardiac cycle at 2.9% and 5.2%. These results further support that the model captures not only the spatial and temporal dynamics of blood flow but also respects core physical laws such as continuity.

### Generalization to unseen cases

In order to evaluate the generalization capability of the model trained solely on semi-idealized geometries, we conducted two experiments without any additional fine-tuning. Indeed, the proposed GNN framework operates entirely on local mesh and flow features rather than relying on global geometry descriptors; therefore, it is essential to assess its ability to adapt to new settings, provided the underlying physics and boundary conditions remain comparable.

**Fig. 14:**
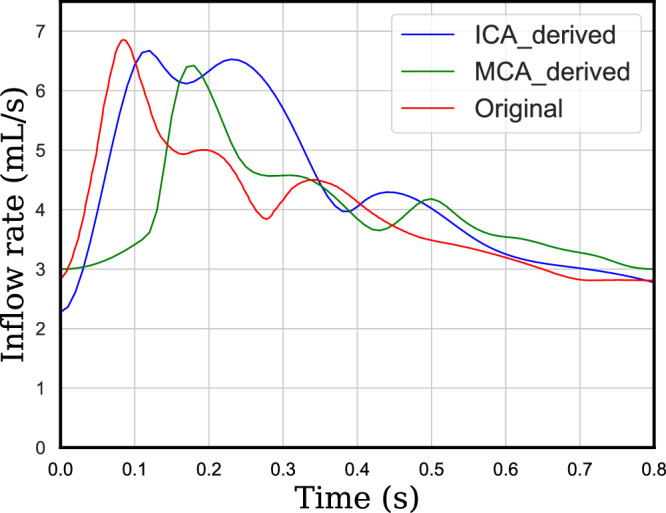
Different inflow rates derived from patient-specific ICA and MCA data^38,39^.

**Fig. 15:**
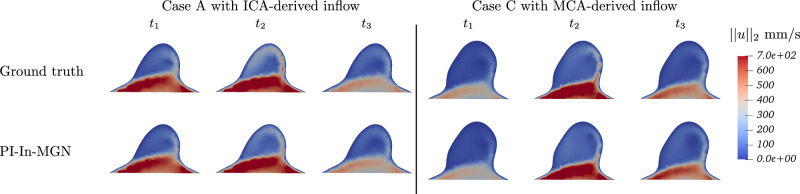
Blood velocity comparison at the aneurysm neck on unseen velocity profiles.

First, we examined the model’s robustness to unseen inflow rates. We ran new CFD simulations on our three test cases using alternative inflow velocity time profiles from ICA and middle cerebral artery (MCA) as well, derived from the literature^38,39^, shown in Fig. 14. When evaluated on these six new inflow conditions (with no retraining), the model achieved a 1-step RMSE of 2.4 and a 50-step RMSE of 14.3. Velocity field comparisons in Fig. 15 confirm that predictions remain close to the ground truth, despite never encountering these inflow profiles during training. This result demonstrates the model’s adaptability to physiologically realistic variations in inflow dynamics, even though it was trained on a single fixed profile.

Second, we tested the model on four zero-shot predictions on out-of-distribution, patient-specific aneurysm geometries. These new cases were randomly selected from our internal database and exhibit a range of morphological variations, including aneurysm location, parent vessel diameter, neck size, aneurysm sac diameter, and centerline length. Their geometrical features are further described in Table 3 and highlight the difference of these cases from the BenchAnXplore training and testing data.

**Table 3:** Zero-shot cases geometrical description with aneurysm location, inlet artery diameter, aneurysm diameter, aneurysm neck diameter, and artery centerline length from inlet to furthest outlet, all in mm

Case	Location	Inlet diameter	Aneurysm diameter	Aneurysm neck diameter	Centerline length
**Case 1**	ICA	4.8	4.5	4.2	49.1
**Case 2**	MCA	2.5	6.0	5.2	23.2
**Case 3**	MCA	2.7	7.8	7.3	21.4
**Case 4**	ICA	4.3	15.0	8.7	65.5
**BenchAnXplore**	ICA	4.0	3–9	3.2	25.6

The BenchAnXplore values are given as a comparison.

**Fig. 16:**
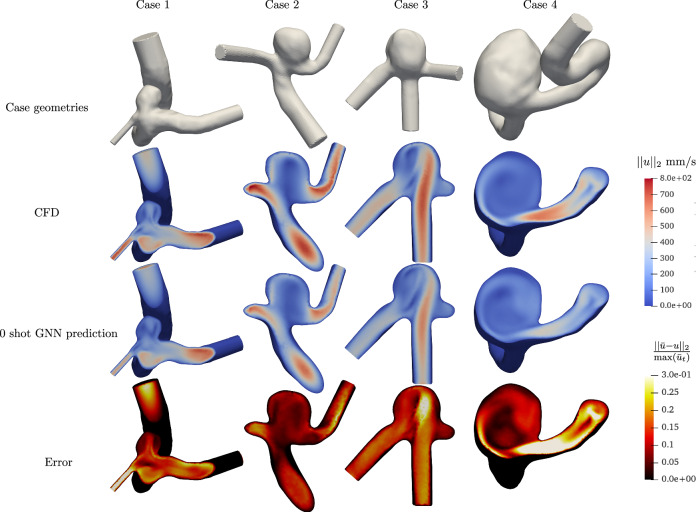
Zero-shot predictions on out-of-distribution patient-specific ICA and MCA aneurysm cases.

Remarkably, the In-PI-MGN model trained only on synthetic semi-idealized geometries was able to reproduce realistic flow behaviors in this complex clinical scenario. While the predicted velocity fields show discrepancies in order-of-magnitude for cases 3 and 4, the predicted velocity fields match the CFD ground truth in spatial distribution in all cases, as shown in Fig. 16.

## Discussion

Our results demonstrate that In-PI-MGN, a physics-constrained GNN, can accurately predict spatio-temporal blood flow fields across a diverse range of 3D aneurysmal geometries. The model maintains close agreement with CFD ground truth while offering significant computational gains. The model can simulate 80 timesteps in under a minute, whereas traditional CFD methods require approximately 1 h for the same task. Even accounting for the 20-h training time, the proposed approach becomes more efficient after just 20 simulations. Including dataset generation, the total model development time amounts to approximately 70 h. This leap in speed could be critical in clinical decision support, especially for time-sensitive or resource-constrained contexts.

Indeed, the ability to produce fast full-field hemodynamic predictions opens new possibilities for simulation-augmented clinical workflows. For instance, rupture risk assessments or device deployment planning could benefit from immediate feedback, without the delays of traditional CFD. We foresee this approach contributing to preoperative planning, follow-up monitoring, and even in-room guidance during interventional neuroradiology.

Our contributions are threefold. First, we tackle the challenge of predicting transient, non-Newtonian 3D blood flow, with the ability to accurately capture the evolution of recirculation and swirling patterns, within complex aneurysmal geometries, an area previously unexplored with GNNs. Second, we introduce two architectural advances: contextual inflow feature enhancement to improve long-range temporal representation, and physics-informed training to promote stability and physical plausibility across time. Third, we release BenchAnXplore, a large-scale benchmark dataset of 105 IA geometries with matched CFD simulations, establishing a new standard for AI-based hemodynamics research.

Prior ML-based approaches for hemodynamic modeling have mostly focused on reduced-order representations, simplified vascular segments, or static features^15,23,25^. Our work departs from this by directly learning spatio-temporal dynamics from full 3D velocity fields across realistic geometries. Leveraging the expressive capabilities of GNNs, our model captures complex flow behavior—including swirling and recirculation—within topologically diverse aneurysm shapes. The integration of physics-informed loss terms and contextual inflow features marks a methodological advance, enabling accurate long-term rollout and reducing error accumulation.

Furthermore, we have confronted our method to out-of-distribution unseen cases with different inflow rates and patient-specific cases, and observed that our model had obtained generalization capabilities and was able to uncover realistic flow patterns with no additional training. These preliminary results highlight the potential of our approach as a foundation for future fine-tuning toward patient-specific clinical applications.

In summary, this work offers a reproducible, extensible framework that brings high-fidelity hemodynamic inference closer to real-time clinical use. By enabling rapid hemodynamic inference with strong physical consistency, this work paves the way for real-time risk assessment, personalized treatment planning, and simulation-augmented clinical workflows. It offers a reproducible and extensible step toward bridging high-fidelity fluid mechanics and deep learning in cerebrovascular medicine.

However, several important limitations remain before clinical deployment can be fully realized. First and foremost, the proposed model is trained on idealized vessel geometries. Clinical routine is characterized by a wide variety of anatomical presentations in terms of size, shape, and orientation. As highlighted by the promising yet degraded performance of our model on out-of-distribution patient-specific cases (e.g., average 50-step relative velocity error on the four cases of 11% compared to 1.6% on the original test set), generalization to clinical settings requires training on representative data. While our current dataset is varied and complex enough to serve as a valuable benchmark for model evaluation, it represents only a first step toward patient-specific hemodynamics prediction. In future work, this will need to be complemented by a larger collection of patient-derived geometries or the generation of more realistic synthetic cases.

Second, our CFD ground-truth simulations were generated using a single, temporally and spatially idealized inflow profile. We have demonstrated the model’s ability to generalize across different inflow time profiles, even when these were not part of the training data. Although these inflow rates are widely used in CFD studies of IAs^40–42^, they remain statistical means extracted from literature^38,39^, and may not reflect true patient-specific variability. It would be highly valuable to extend this approach to include clinically acquired Doppler ultrasound or 4D Flow MRI inflow profiles. Additionally, all inlet profiles in this study are prescribed as idealized parabolic flows, which—while common^41–43^, omit higher-order effects captured in more realistic Womersley profiles^40^. Fortunately, previous studies have shown that aneurysmal flow patterns remain robust across such inlet assumptions when appropriate domain lengths are used^44^. Nonetheless, we aim to integrate 4D Flow MRI-derived boundary conditions in future work^45^, both for fine-tuning and evaluating our model under more realistic conditions.

Lastly, we acknowledge that while hemodynamic quantities such as WSS and OSI are commonly studied, a biomarker strongly correlated with IA rupture risk remains elusive. The interpretation of these biomarkers, including proposed thresholds for risk stratification, continues to be debated within the scientific and medical communities. While the primary objective of this work is not direct rupture prediction, it is important to clarify that our model outputs are intended to serve as high-fidelity, physics-consistent inputs to further risk analysis, not as standalone diagnostic tools. We see this as a step toward augmenting clinical decision-making by providing accessible, real-time flow field estimation, which can be combined with other risk factors to support informed diagnostic.

Building on this foundation, future work may explore advanced architectures such as multigrid GNNs^46^, which enable hierarchical information flow across mesh scales, and attention-based GNNs, which enhance the model’s ability to prioritize key flow structures. These approaches could enable higher-resolution predictions while preserving computational efficiency, further advancing AI-driven hemodynamic modeling. Moreover, while we demonstrate zero-shot generalization on both unseen inflow profiles and a fully patient-specific case, we emphasize the need for further validation on larger, more heterogeneous clinical datasets to support further research.

## Methods

### Graph neural networks

GNNs are a class of neural networks working on sets of vertices and edges. Through different deep learning techniques such as convolution (Graph Convolutional Networks)^47^, attention (Graph Attention Networks)^48^, or recurrent layers (Graph Recurrent Networks)^49^, they exploit message passing mechanisms to learn the relations between neighboring nodes, and have shown great performances in many areas of physics^19,21,50–52^.

Solving complex systems of partial differential equations, such as the Navier-Stokes equations, often requires mesh-based discretisation of the computational domain. These meshes are typically adapted to enhance numerical accuracy in critical regions while minimizing computational cost in less challenging areas^53^. However, traditional deep-learning architectures, such as convolutional, recurrent, or fully connected layers, struggle to effectively handle the anisotropic and irregular nature of meshes. They either fail to capture local dependencies properly or do so at a prohibitive computational expense. Building on the founding that the deep-learning toolbox layers did not offer the possibility of learning arbitrary relations between elements of a set with relational structure, such as a graph, Battaglia et al.^54^ introduced the Graph Network (GN) layer, aiming to create a general framework for graph computation. Inspired from Message Passing Neural Networks^55^ among others, this layer is designed to take a graph as input, process the graph with any subsequent model, such as a neural network, but not necessarily, and return a graph, using the nodes as entities with features, edges as relations between entities, and global attributes over the whole graph.

We note an undirected graph *G* = (*V*, *E*) with *V* a set of vertices with $V={\{{v}_{i}\}}_{i\in {N}^{v}}$ vertices attributes, and *E* a set of edges with *e*_*ij*_ linking vertices with indexes *i*, *j*.

**Fig. 17:**
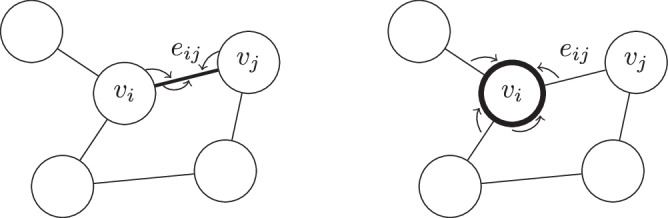
Graph network updating edges (left) and nodes (right).

The GN updates edge attributes with an edge-update function *φ*_*E*_, gathers a vertex neighborhood information by aggregating the updated edges connected to it with an aggregation function *ρ*, and updates the vertex with a vertex-update function *φ*_*V*_ (Fig. 17). Because the update functions are shared over every node and edge, GNs are adapted to learn local physical behavior over every relation in the graph. We purposefully left out global graph attributes here to avoid unnecessary complications of the framework, as we will not be using them.

1 $$\begin{array}{rcl}{\widetilde{e}}_{ij} & = & {\varphi }_{E}({e}_{ij},{v}_{i},{v}_{j})\\ {\widetilde{e}}_{i} & = & \rho ({\{{\widetilde{e}}_{ij}\}}_{j})\\ {\widetilde{v}}_{i} & = & {\varphi }_{V}({v}_{i},{\widetilde{e}}_{i})\end{array}$$

with $\widetilde{{e}_{ij}}$ being the updated edge attributes from the initial edge attributes and the connected vertices attributes, ${\widetilde{e}}_{i}$ being the aggregated information of all edges linked to vertex *i*, and ${\widetilde{v}}_{i}$ being the updated vertex attributes from the initial vertex attributes and the aggregated neighborhood information.

### MeshGraphNet—GNNs for physics simulations

**Fig. 18:**
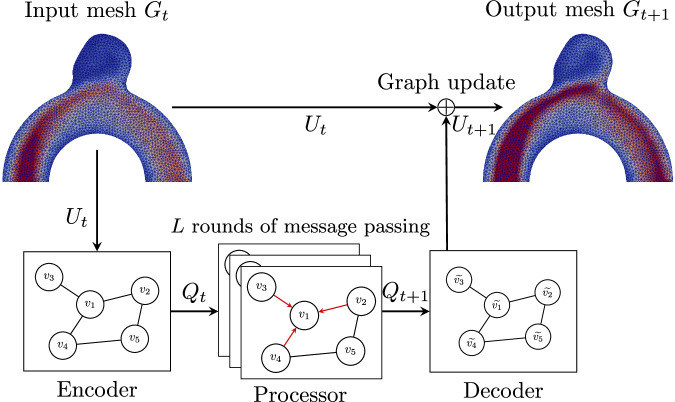
Encode-process-decode (EPD) architecture^17^ for velocity *U*_*t*+1_ prediction from *U*_*t*_.

**Fig. 19:**
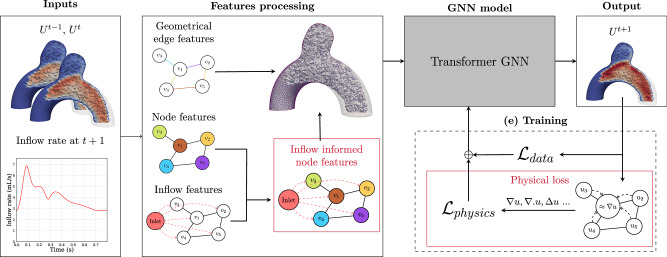
Model architecture - the input meshes and inflow data are exploited to create a feature graph, from which our GNN model can predict the next step mesh, taking into account physical and data losses combined during model training.

Numerical physics simulations like CFD relies on meshes. Pfaff et al.^17^ took advantage of this data structure and built a GNN model using physical meshes as inputs, designed for next-step prediction in physical problems such as flow field prediction around a cylinder or a wing profile. With the mesh information at *t* (and potentially *t* − 1), the trained network can predict the physical fields at *t* + 1. By inferring on its own predictions, the network can predict a whole simulation with only a few timesteps to infer from; the MeshGraphNet (MGN) model has achieved state-of-the-art results, being able to predict the temporal evolution of these systems during significant time periods while remaining coherent with the physical simulations. The MGN model learns the system’s forward dynamics with an encode-process-decode architecture (Fig. 18), using multilayer perceptrons (MLPs) Fig. 19.

#### Encoder

The encoder extracts all input features for nodes and edges from the graph *G*_*t*_, which is derived from the mesh at time *t*. These features include physical properties such as velocity, pressure, or stress, as well as geometrical attributes like edge lengths and node positions. The node and edge features are respectively encoded into latent spaces by the encoder MLPs *ϵ*_*V*_ and *ϵ*_*E*_ into latent spaces of size 128, as graph nodes *v*_*i*_ and edges *e*_*ij*_, to constitute the latent space graph *Q*_*t*_.

#### Processor

The processor then performs rounds of message passing by applying *L* several successive GN layers. For each round of message passing, each node of the graph sends messages to its neighbors, which consist of the encoded and processed node’s and edge’s feature information. As in the GN layer described in Subsection GNNs, the neighbors aggregate and process these messages to gain a collective understanding of their local neighborhood and to update their own feature representation. This allows them to incorporate the new information received from their neighbors and refine their own states. Each of these GN layers has update functions *φ*_*E*_ and *φ*_*V*_, respectively, for edges and nodes, as described in Equation (1). These functions are MLPs as well, with different sets of parameters for each GN layer, and the edge aggregation function *ρ* is an element-wise summation. Performing several consecutive rounds of message passing allows the GNN to infer the evolution of the graph with information circulating over the whole domain, and to return an updated latent space graph *Q*_*t*+1_ with updated node and edge latent vectors ${\widetilde{v}}_{i}$ and ${\widetilde{e}}_{ij}$.

#### Decoder

Finally, the decoder translates the processed node vectors contained in the latent space updated graph *Q*_*t*+1_ with a final MLP *δ*_*V*_ to a physical update, which is added to the input mesh in order to get the next-step physical mesh *G*_*t*+1_ with updated physical fields.

All the underlying processing and updating functions here are ReLU-activated 2 hidden-layer MPLs. The model learns the physical behavior by minimizing the differences between its predictions and the ground truth data computed with a numerical solver, with a data loss being the mean square error, for a given physical quantity *a*:

2 $${{\mathcal{L}}}_{data}=\mathop{\mathrm{mean}}\limits_{v\in V}\parallel {a}_{v}-\widetilde{{a}_{v}}| {| }_{2}^{2}$$

with *a*_*v*_ being the ground truth and $\widetilde{{a}_{v}}$ the prediction at node *v*.

### Hemodynamics simulations

The MeshGraphNet model has demonstrated excellent performance in predicting flow in 2D settings for benchmark fluid dynamics problems, such as incompressible steady flow around a cylinder and compressible flow around an airfoil. However, its ability to generalize to blood flow predictions in a three-dimensional environment remains an unexplored frontier and requires further investigation.

The CFD numerical framework used in hemodynamics simulations provide fully resolved velocity *u* and pressure *p* fields on the whole 3D domain by solving the mixed formulation of the transient incompressible Navier-Stokes equations given by:

3 $$\begin{array}{rcl}\rho ({\partial }_{t}u+u\cdot \nabla u) & = & -\nabla p+\nabla \cdot (2\mu \epsilon (u))\\ \nabla \cdot u & = & 0\end{array}$$

with *ρ* the fluid density, *ϵ*(*u*) the strain rate tensor and *μ* the fluid viscosity. Blood exhibits a non-Newtonian rheology, a shear-thinning behavior that can be described with constitutive models such as the Casson or the Carreau–Yasuda law^56^:

4 $$\mu (\dot{\gamma })={\mu }_{\infty }+({\mu }_{0}-{\mu }_{\infty }){\left(1+{(\tau \mathop{\gamma }\limits^{\cdot })}^{a}\right)}^{\frac{n-1}{a}}$$

with the relaxation time *τ*, power indices *n* and *a*, and asymptotic values of the effective viscosity $\mu (\dot{\gamma })$, *μ*_0_, and *μ*_*∞*_, at zero and infinite shear-rates, respectively, being known fluid constants.

At the inlet of the fluid domain, we impose a physiologically realistic pulsatile mass flow profile corresponding to one full cardiac cycle, derived from ICA measurements as reported in the literature^57^. The velocity profile is applied as a fully developed parabolic inflow condition. To ensure smooth initialization of the simulation, the cardiac waveform is preceded by a 0.2 s linear ramp-up. The simulation spans two full cardiac cycles of 0.8 s each, with the first cycle serving as transient initialization. This waveform corresponds to a cardiac cycle of 0.8 s and was scaled to produce a peak systolic flow rate of approximately 6 mL/s, which, given the 4 mm vessel diameter, yields a peak Reynolds number of approximately 400. This flow regime is consistent with reported physiological conditions in the internal carotid artery^34,36,38,57^. Note that only the second cycle is retained for analysis to ensure periodic flow has been reached. At the outlet, we apply a resistive boundary condition to emulate the downstream vasculature. The resistance is tuned to maintain a physiological pressure variation of approximately 40 mmHg over the cardiac cycle, consistent with clinical observations for ICA outflow and guided by known systolic and diastolic flow rates. The vessel walls are modeled as rigid with a no-slip boundary condition, hence zero velocity is imposed at all wall-adjacent nodes. This is a standard assumption in many CFD studies of IAs, particularly when the primary interest lies in evaluating flow patterns and shear-related biomarkers rather than wall compliance.

Several quantities of interest have been identified for risk assessment and treatment decisions for unruptured IAs. Obtaining these quantities by post-processing the simulated velocity fields inside the aneurysm is of great interest for clinical routine, and is a key feature expected from CFD frameworks for hemodynamics. Among these hemodynamical quantities is the WSS, defined here as:

5 $$\tau =n\times [(\sigma \cdot n)\times n]=\sigma \cdot n-[(\sigma \cdot n)\cdot n]n$$

where ***n*** is the unit normal vector at the wall and ***σ*** is the Cauchy stress tensor defined as *σ* = − *pI* + *μ*(∇ *u* + ∇^*T*^*u*).

This framework unveils a higher level of complexity compared to benchmark fluid dynamics cases on which MGN was evaluated. The shift to 3D adds significant intricacy to the observed flow patterns and increases the size of the meshes. Additionally, the geometries at hand in a medical context are derived from real patients, making them inherently more intricate and variable compared to synthetic or benchmark models. Moreover, blood rheology can be considered in hemodynamics simulations, needing to account for non-Newtonian fluid properties and their effects on flow behavior. This adds another layer of complexity as blood exhibits varying viscosity under different flow conditions, as shown in Equation (4). Finally, the inflow conditions in blood flow studies follow a pulsatile cardiac cycle, which introduces time-dependent variations that must be accurately captured to understand the hemodynamics accurately. This pulsatile nature requires a deeper understanding of hemodynamics by the model to simulate the rhythmic changes in blood flow and pressure.

For a GNN-based CFD surrogate approach to be justified, the GNN counterpart to traditional numerical solvers needs to produce local physical spatio-temporal fields in a relatively thin margin of error from the CFD ground truth outputs, with a significant reduction in computing time.

### Dataset generation

For each of the 105 semi-idealized aneurysm geometries, numerical fluid simulations were performed following the general fluid mechanics problem formulated in the previous subsection, with a 0.001 s timestep. All the geometries hemodynamics were simulated using the velocity profile derived from^57^. The simulations ran on an in-house finite-element C++ based library by solving a mixed formulation of the incompressible Navier–Stokes equations (Equation (3)) using a variational multiscale method, as described by Hachem et al.^58^.

The dataset meshes were generated with refined boundary layers around the artery walls to accurately compute velocity gradients during the CFD simulations. The resulting meshes are composed of around 200 k nodes each, which translates to about 1 M elements.

In order to build a reproducible experiment with a versatile dataset, we derived a lighter version of the dataset, allowing us to test many configurations with reasonable computational resources and time. This lighter dataset was generated using an automated pipeline. Starting from the high-resolution CFD meshes with refined boundary layers, we extracted the outer surface of each vascular geometry and re-meshed it using isotropic triangular elements. These surfaces were then used to generate volumetric meshes consisting of unstructured tetrahedral elements with uniform resolution, using an average target element size of 0.3 mm. This mesh size was selected based on a trade-off between physical fidelity and computational efficiency, after experimenting with multiple mesh resolutions, ranging from 0.15 to 0.4 mm.

No boundary layer refinement was applied in the light dataset, as our aim was to reduce memory requirements and allow rapid GNN training iterations. To populate these lighter meshes with flow data, we applied linear interpolation of the full-resolution CFD velocity and pressure fields onto the new node sets. To validate the accuracy of this interpolation procedure, we performed round-trip interpolation experiments: interpolated flow fields from the fine mesh were mapped onto the coarse mesh and then back onto the original fine mesh. The resulting discrepancies in both velocity and pressure fields remained below a few percent across the domain, confirming the adequacy of the remeshing and interpolation steps for surrogate modeling.

With this framework, meshes with under 25 k nodes and 120 k elements were automatically generated, resulting in a dataset of 105 aneurysm geometries, with 80 simulated timeframes for each of these cases, with a 0.01 s timestep. The obtained dataset, referred to as **BenchAnXplore** dataset, is now made available as a benchmark case for AI IAs applications.

By working on this lighter dataset, we were able to increase our model size, train much faster, and run several experiments in a reasonable time; training one epoch of our models on the original dataset took around 40 times longer than on the lighter dataset (respectively 20 h and 30 min on a NVIDIA A100 GPU).

### Node feature enhancement with contextual inflow information—In-GNN

We designed our model using the MeshGraphNet (MGN) model^17^ presented in Subsection MeshGraphNet—GNNs for physics simulations as a baseline to build upon, and to assess our obtained results. This baseline model uses the velocity field at time *t* as input, and returns the velocity update for time *t* + 1. However, several challenges arise compared to prior GNN applications that have largely focused on 2D scenarios, steady-state 3D flows, or external flow problems around simpler geometries. This work directly confronts the formidable challenges posed by the highly non-linear, pulsatile nature of blood flow within the 3D complex, irregular, and patient-derived geometries of aneurysms. Indeed, the dynamics of the whole domain are deeply linked with the pulsatile nature of the inlet flow, but information from the inflow would need many rounds of message passing to reach every node of our mesh with the raw MGN architecture, fed on velocity data. Following the lines in Gladstone et al.^21^, who faced the problem of long-range interaction in a time-dependent mechanical problem, we fed our network with additional information by adding statistical information about the inflow acceleration at *t* + 1 to every node of the mesh, as the boundary conditions at the inlet are known beforehand.

This allowed the whole domain to grasp the evolution of the inflow dynamics whilst trying to predict the velocity at *t* + 1, without adding layers of message passing, which would have made the model heavier. Moreover, we also added the acceleration between timesteps *t* − 1 and *t* as a node feature to give more local temporal history to the model.

For the baseline MGN model, the node feature vector is composed of the following concatenated quantities:

- Velocity (3 components).
- Spatial position (3 components).
- Timestamp (1 component).
- Node type (1-hot encoded: inlet, wall, interior, outlet).

For our model, we enrich this vector by adding:

- Local acceleration computed as the velocity difference between *t* and *t* + 1 (3 components).
- Inflow context vector: mean, minimum, and maximum velocity magnitude of inlet nodes at *t* + 1 (3 components).

The edge feature vector, consistent across all models, contains:

- The relative coordinate vector between connected nodes (3 components).
- The Euclidean distance (1 component).

### Physically-constrained GNN

Although the model is trained on datasets generated by an in-house numerical solver that computes solutions to the Navier–Stokes equations (see Subsection Dataset), the integration of physical knowledge into the training process can enhance the accuracy and reliability of its predictions. This approach aligns with the principles outlined in physics-informed machine learning, which has been shown to improve predictive performance by embedding domain-specific laws and constraints directly into the learning framework^59^.

Moreover, a data-only-based learning approach can lack robustness to out-of-distribution cases. To enhance the performance of our model, we added a physics-based regularization through the addition of a more physically relevant loss (PI-GNN) by Suk et al.^16^.

Thus, we approximated differentiable operators in a discrete vector field using finite differences over the neighborhood of nodes, thereby preventing training slowdowns caused by gradient computations. These gradients are computed locally within each cell neighborhood on the mesh, which helps preserve topological consistency even in geometrically complex zones.

With *u*_*i*_ the velocity vector at node *n*_*i*_ ∈ *N*, ${{\mathcal{N}}}_{i}$ the collection of its direct neighbors and *x*_*i*_ its position, we can approximate the gradient of *u*_*i*_ with:

6 $$\nabla {u}_{i}\approx \frac{1}{{{\mathcal{N}}}_{i}}\mathop{\sum }\limits_{j\in {{\mathcal{N}}}_{i}}\frac{({u}_{j}-{u}_{i}){({x}_{j}-{x}_{i})}^{T}}{| | {x}_{j}-{x}_{i}| {| }_{2}^{2}}$$

Following the same method, we can write the conservation of mass using the discrete approximation of the divergence:

7 $$\nabla \cdot {u}_{i}=\frac{\partial {u}_{i,x}}{\partial x}+\frac{\partial {u}_{i,y}}{\partial y}+\frac{\partial {u}_{i,z}}{\partial z}=0\,\,{\rm{thus}}\,\,\nabla \cdot {u}_{i}\approx \frac{1}{{{\mathcal{N}}}_{i}}\mathop{\sum }\limits_{j\in {{\mathcal{N}}}_{i}}\frac{({u}_{j}-{u}_{i})\cdot ({x}_{j}-{x}_{i})}{| | {x}_{j}-{x}_{i}| {| }_{2}^{2}}$$

Furthermore, we evaluate the convective term *u* ⋅ ∇ *u* and the viscous term *μΔu* using the following approximation of the Laplacian operator:

8 $$\Delta u\approx \frac{1}{{{\mathcal{N}}}_{i}}\mathop{\sum }\limits_{j\in {{\mathcal{N}}}_{i}}\frac{(\nabla {u}_{j}-\nabla {u}_{i}){({x}_{j}-{x}_{i})}^{T}}{| | {x}_{j}-{x}_{i}| {| }_{2}^{2}}$$

Using these approximations, we developed a physical loss function to augment our training by incorporating not only the discrepancies between predicted and ground truth velocities, but also a deeper understanding of the fundamental principles of fluid mechanics. This approach enables us to calculate a continuity loss based on the conservation of mass, as well as convective and viscous losses, which quantify the errors in the convective and viscous terms, respectively (Equation (3)):

9 $${{\mathcal{L}}}_{continuity}=\mathop{\mathrm{mean}}\limits_{v\in V}|{(\nabla \cdot u)}_{v}|$$

10 $${{\mathcal{L}}}_{convection}=\mathop{\mathrm{mean}}\limits_{v\in V}\parallel \rho {u}_{v}\nabla {u}_{v}-\rho \widetilde{{u}_{v}}\nabla \widetilde{{u}_{v}}| {| }_{2}^{2}$$

11 $${{\mathcal{L}}}_{viscous}=\mathop{\mathrm{mean}}\limits_{v\in V}\parallel \mu \Delta {u}_{v}-\mu \Delta \widetilde{{u}_{v}}| {| }_{2}^{2}$$

To mitigate the importance of each loss term during training, we combined the data and physical terms in a properly tuned weighted loss, as following:

12 $${{\mathcal{L}}}_{PI}={{\mathcal{L}}}_{data}+\alpha {{\mathcal{L}}}_{continuity}+\beta {{\mathcal{L}}}_{convection}+\gamma {{\mathcal{L}}}_{viscosity}$$

with *α*, *β*, and *γ* being scaling factors. We tuned our weighted loss by evaluating the physical loss terms during the first epochs of training the data-only equivalent models, to adjust the scaling factors in order to get comparable scales for each contributor of the global loss. The data loss term was given a weight of 0.5, while the remaining physics-informed terms were equally distributed over the remaining 0.5. While the continuity term stabilizes after only one epoch, the convection and viscosity losses decrease steadily during the whole training alongside the data loss, providing a faster model convergence.

### Evaluation metrics

To compare the performances of our models, we computed the L2 norm of the velocity error, and with this error, the Root Mean Square Error (RMSE) averaged across case geometry. We used the 1-RMSE to evaluate the next-step predictions of our model, as well as the 50-RMSE on a rollout prediction to determine the robustness of the model on full trajectory predictions, as formulated in Equation (13). We displayed these errors, averaged on the test set, in Table 1, as well as the standard deviation of the 50-step RMSE to evaluate the stability of the models on different test cases. These errors are in mm/s, and the values reported in Table 1 are absolute errors, not normalized by maximum velocity.

13 $$\begin{array}{rcl}1-{\rm{RMSE}} & = & \sqrt{\frac{\left.{\sum }_{i}^{N}{({y}_{i}^{t}-f{({x}^{t-1})}_{i})}^{2}\right)}{N}}\\ T-{\rm{RMSE}} & = & \frac{1}{T}\mathop{\sum }\limits_{t}^{T}\sqrt{\frac{\left.{\sum }_{i}^{N}{({y}_{i}^{t}-{f}^{t}{({x}^{0})}_{i})}^{2}\right)}{N}}\end{array}$$

**Fig. 20:**
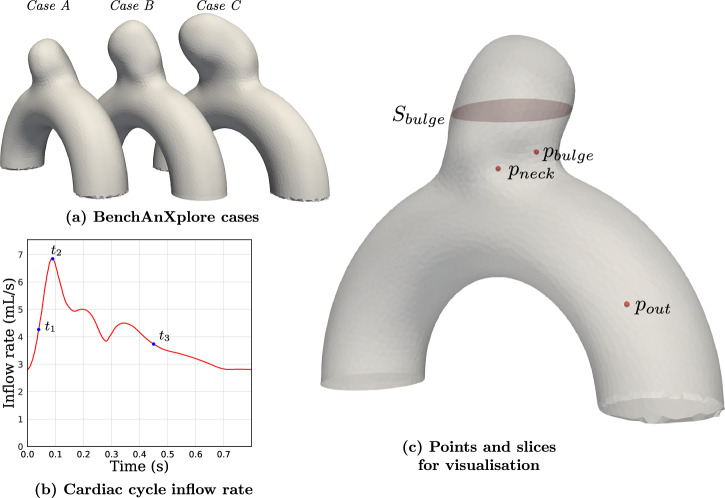
Evaluation cases, inflow conditions, and sampling locations. **a** Cases selected from the test dataset for detailed analysis (cases A–C). **b** Prescribed pulsatile inflow rate over one cardiac cycle, with three representative time instants (t1t_1t1, t2t_2t2, t3t_3t3) selected for evaluation. **c** Slice and point locations used for velocity data extraction, including the bulge cross-section and the points ${p}_{{neck},}\,{p}_{bulge}$, and ${p}_{{out}}$.

Three representative cases were randomly chosen from the 10 test cases while guaranteeing coverage of three bulge sizes. Three key timesteps were also selected along the cardiac cycle, as shown in Fig. 20, to represent the systolic, peak systole, and diastolic phases. We computed WSS as well as time-averaged WSS (TAWSS) as defined in Methods to further analyze the prediction capability of the models needed for risk assessment. All WSS-based quantities were derived directly from the velocity fields and were not interpolated from the finer cases. We also plotted the aneurysm neck plane to show the velocity of the flow entering and exiting the bulge, as well as on three selected points along the full cardiac cycle to show the velocity evolution inside the neck, the outflow artery and close to the bulge wall, as shown in Fig. 20. The mentioned velocity and WSS errors are normalized by the ground truth maximum at a given timestep; because of the highly dynamic nature of the flow, normalizing by the overall maximum value would highly reduce the error on every timestep far from the peak systole.

## Data Availability

The datasets used and analyzed during the current study are available at the following link: https://storage.googleapis.com/large-physics-model/datasets/aneurysm/coarse_03_dataset.zip. The custom code used for data generation and visualization are relying solely on open-source Python libraries and are available from the corresponding author upon reasonable request as well.

## Code availability

The underlying code for this study are available from the corresponding author upon reasonable request. The custom code used for data generation and visualization are relying solely on open-source Python libraries and are available from the corresponding author upon reasonable request as well.
